# Conservative treatment for urinary fistula following ileal conduit urinary diversion: a simple method

**DOI:** 10.1186/s12894-019-0564-3

**Published:** 2019-12-10

**Authors:** Yun-lin Ye, Hai-tao Liang, Lei Tan, Xia Zheng, Dan Xiong, Kang-hua Xiao, Zi-ke Qin

**Affiliations:** 1 Department of Urology, Sun Yat-sen University Cancer Center, State Key Laboratory of Oncology in South China, Collaborative Innovation Center for Cancer Medicine, Guangzhou, 510060 Guangdong China; 2 Department of Urology, the First Affiliated Hospital of Sun Yat-sen University, Guangzhou, 510080 Guangdong China; 3 Medical Laboratory of The Third affiliated Hospital of Shenzhen University, Shenzhen, 518000 Guangdong China

**Keywords:** Urinary fistula, Ileal conduit, Negative pressure system, Intra-conduit, Conservative treatment

## Abstract

**Background:**

The presence of urinary fistula after ileal conduit urinary diversion is a challenging complication, and this study investigated the role of the intra-conduit negative pressure system (NPS) in the presence of urinary fistula following ileal conduit (IC) urinary diversion as a conservative treatment.

**Methods:**

Using the intra-conduit NPS, a minor drainage tube was placed within a silicon tube to suck urine from the conduit with consistent negative pressure. Patients with urinary fistula following IC from August 2012 to July 2017 were recorded, and the clinical characteristics and outcome were retrospectively analyzed.

**Results:**

The intra-conduit NPS was used as a primarily conservative treatment for 13 patients who suffered from urinary fistula and presented with a large amount of abdominal/pelvic drainage without other significant morbidities. The median age was 60 years old (42–74 years), and 7patients were male. The median duration between the IC operation and the presence of urinary fistula was 15 days (2–28 days), and elevated creatinine levels were detected in the abdominal/pelvic drainage with a median level of 2114 μmol/L (636–388 μmol/L). A significant decrease in abdominal/pelvic drainage was identified in 12 patients. The median time that the NPS was used was 9 days (7–11 days). The other patient did not show any improvements after 2 days of observation and then underwent open surgery. With ureteral stenting, 2 abdominal drainage tubes and the intra-conduit NPS were placed during operation, no urine leakage was observed in the abdominal/pelvic field, and the patient was cured in 9 days. With a median follow-up of 22 months, no fistula recurrence or hydronephrosis was detected.

**Conclusion:**

The intra-conduit negative pressure system is a feasible and promising way to cure urinary fistula following ileal conduit urinary diversion. Because this procedure is a mini-invasive and simple approach, it might represent an alternative in selected patients.

## Background

Cystectomy and urinary diversion are some of the most complicated procedures in urological operations, with a nearly 40% perioperative morbidity rate. The presence of urinary fistula after ileal conduit urinary diversion is rare, but the management of this complication is challenging [1–4]. Although ureteroenteric anastomosis and conduit closure both carry the risk of urine leakage, management is simple as follows: evaluate and ensure urinary fistula, drain the urine, and then repair the fistula actively or conservatively [4–8]. Compared to surgical approaches, retrograde stent placement and nephrostomy are more common mini-invasive approaches than surgical approaches for addressing the presence of urinary fistula following ileal conduit urinary diversion [8–11]. A contrastographic study before nephrostomy can be used to comprehensively evaluate abdominal/pelvic ureter and ureterointestinal anastomoses. However, sometimes urine cannot be completely drained, and a balloon is used to obstruct the ureter during nephrostomy. Although completeness has been favorable in recent studies, it is a complicated mini-invasion procedure in clinical practice.

Negative pressure system (NPS) drainage has generally been used to cure complicated wounds [12–14]. In a sparse number of reports, NPS drainage has also been revealed as a promising outcome in the management of urinary fistula [15–17]. Therefore, we tried to use an intra-conduit NPS as a conservative way to cure patients who had suffered from urine leakage after ileal conduit urinary diversion since 2012. Initially, the NPS was only recommended for patients with good performance as an alternative for retrograde stenting or nephrostomy. We now report our preliminary experiences using the intra-conduit NPS to address the presence of urinary fistula after ileal conduit urinary diversion.

## Methods

Patients who underwent ileal conduit urinary diversion in our center were retrospectively reviewed from August 2012 to July 2017. Urine leakage was diagnosed by imaging and/or the amount of abdominal/pelvic drainage reported in drainage creatinine studies. For patients who did not present with significant abdominal infection or other severe morbidities, the intra-conduit negative pressure system was set as the conservative treatment. If this treatment did not work within 2 days, subsequent treatments such as the retrograde placement of ureter stents, nephrostomy or open surgery would be performed. Informed contest was confirmed after a comprehensive consultation.

**Fig. 1:**
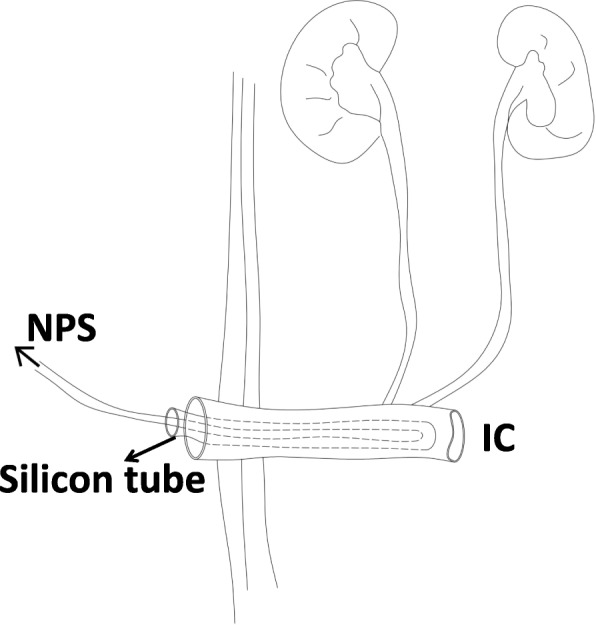
Sketch of the intra-conduit negative pressure system NPS: negative pressure system; IC: ileal conduit.

A sterile silicon tube (F18 abdominal drainage tube) with lateral holes was reset into the conduit in patients who had this tube removed after the operation. Then, a mini-plastic tube (F12 stomach tube) with lateral holes was also placed into the silicon tube and was tied to a negative pressure system (Figure 1). During this process, if the ureteral stents had not been previously removed, they were now kept, and the silicon tube was gently placed into the ileal conduit (Additional file 1: Figure S1). When the negative pressure system had a pressure of 20–25 cmH_2_O, urine was drained out continuously, and the abdominal/pelvic drainage decreased significantly. Most of the time, the NPS was supplied by the central negative pressure system; when the patients were released from the hospital bed, a negative pressure machine with a battery was administered. This process could be accomplished at the bedside and would work for approximately one week, where no urine leakage was detected by clinical evaluation as characterized by the dose of urine, the amount of abdominal drainage, the creatinine drainage level, patient complaints, etc.

The perioperative clinical features were retrospectively analyzed, and the final follow-up was completed in October 2018. Survival status was recorded, and imaging of the upper urinary tract was retrieved during follow-up of patients with primary cancer.

## Results

During these 5 years, 446 cases of ileal conduit urinary diversion were completed following radical cystectomy or pelvic exenteration in our center. All ureterointestinal anastomoses were performed using Bricker ureteral implantation, and 18 patients suffered from urine leakage 30 days after this difficult procedure. Of these patients, 13 presented with a high amount of abdominal/pelvic drainage and decreased urine output from the ileal conduit without significantly severe morbidities. An intra-conduit negative pressure system was first chosen as the conservative treatment for these patients.

The median age was 60 years old (42–74 years), and 7patients were male. The primary diagnosis was bladder cancer in 11 patients. The median interval between IC and the diagnosis of urinary fistula was 15 days (2–28 days), and elevated creatinine levels were detected in the abdominal/pelvic drainage with a median level of 2114 μmol/L (636-3852 μmol/L). Abdominal X-ray was performed in 4 patients to identify the locations of the ureteric stents, and abdominal/pelvic contrast-enhanced CT was performed in 2 patients without positive findings. An intravenous pyelogram confirmed 1 ureterointestinal anastomotic fistula, but this method was not used in the first 2 weeks after the operation. Additionally, an ileal conduit contrastographic study confirmed 2 conduit fistulas. When the intra-conduit NPS was used as the primary treatment, a significant decrease in abdominal/pelvic drainage was identified in 12 patients. For these patients, the median time that the NPS was used was9 days (7–11 days), and then the fistula was cured (Table 1).

**Table 1:** Clinical characteristics of patients underwent intra-conduit negative pressure system

No.	Age (year)	Primary cancer	Major operation	Interval (day)^a^	Status of Ureter stent	Time of NPS (day)	Outcome
1	< 60	Epithelioid trophoblastic tumor of the uterine	Pelvic exenteration	21	Kept	9	Cured
2	< 60	Cervical cancer	Pelvic exenteration	28	Moved	10	Cured
3	< 60	Bladder Cancer	Radical cystectomy	2	Kept	7	Cured
4	< 60	Bladder Cancer	Radical cystectomy	3	Kept	7	Cured
5	< 60	Bladder Cancer	Radical cystectomy	15	Kept	9	Cured
6	> 60	Bladder Cancer	Radical cystectomy	24	Moved	10	Cured
7	> 60	Bladder Cancer	Radical cystectomy	8	Kept	9	Cured
8	> 60	Bladder Cancer	Radical cystectomy	3	Kept	7	Cured
9	< 60	Bladder Cancer	Radical cystectomy	14	Kept	9	Cured
10	> 60	Bladder Cancer	Radical cystectomy	18	Moved	10	Cured
11	> 60	Bladder Cancer	Radical cystectomy	17	Moved	10	Cured
12	> 60	Bladder Cancer	Radical cystectomy	15	Moved	2	Failed^bb^
13	> 60	Bladder Cancer	Radical cystectomy	4	Kept	11	Cured

*NPS* Negative pressure system

^a^Time between fistula and surgery

^bb^Transformed to surgical approach

The other patient showed no improvement after 2 days of observation and then underwent open surgery for the combination of consistent ileus and suspicious abdominal infection. Preoperative contrast imaging showed a significant leak at the location of the distal conduit. During the reoperation, stickiness made it difficult to repair a 1-cm hole linked to the left ureterointestinal anastomosis. During this operation, we found that a sticky belt pressed over the distal third of the ileal conduit, and the silicon tube of the NPS did not cross this stricture to suck urine out completely. Double ureter stents and 2 abdominal drainage tubes were placed, and a new silicon tube was placed to the end of the ileal conduit. No urine leaked into the abdominal/pelvic field, and the patient was cured 9 days after the operation.

With a median follow-up of 22 months (16–46 months), 1 patient died of cervical cancer 41 months after NPS treatment. No hydronephrosis was detected, and no recurrence of fistula occurred.

## Discussion

Urinary fistula following ileal conduit urinary diversion is rather rare, but it is associated with severe comorbidities such as abdominal infection, ileus, and metabolism impairment. Treatment is challenging, especially when it is administered following complicated pelvic organ resection and urinary diversion [6, 8, 9]. For these fragile patients, the management of urinary fistula should be as minimally invasive as possible. A surgical approach is usually avoided due to postoperative complications and stickiness. In recent years, several approaches including a retrograde ureteral approach and percutaneous nephrostomy have been developed in the field of endourology to address this complicated consequence, but neither of these two options is easy to accomplish [18–21].

In this cohort, most (12/13) patients with urinary fistulas following ileal conduit urinary diversion were cured with the intra-conduit NPS, which was a time-consuming (7–11 days) but an extremely simple, safe and mini-invasive method. For these selected patients, the use of the intra-conduit NPS as a conservative treatment was compatible and tolerated, as this bedside process was nearly noninvasive. It was easy to evaluate the effect of the NPS via the decreases in abdominal/pelvic drainage and the normalized creatinine level. For the patient who experienced failure of NPS, we found that the ileal conduit was pressed by a sticky belt during the operation so that the silicon tube could not reach the end of the conduit. This may have resulted in the inability of urine to be successfully and completely suctioned. When the silicon tube was successfully placed during the second operation, the NPS worked efficiently after the operation. In fact, most urine leakage following ileal conduit urinary diversion was due to ureteroenteric anastomosis and conduit closure, so the drainage of urine out of the conduit was critical to cure urine leakage, and the intra-conduit NPS might be a good procedure to accomplish this purpose.

In recent years, an increasing number of studies have demonstrated that endourology approaches are feasible for dealing with upper urinary tract lesions. Olson Land colleagues reported that the success rate was approximately 74%(40/54) during retrograde endourological management of upper urinary tract abnormalities [10]. An antegrade percutaneous flexible endoscopic approach also demonstrated favorable outcomes [11].

In clinical practice, percutaneous nephrostomy is feasible and relatively safe for ureteroenteric anastomosis stricture. However, for patients with urine leakage following ileal conduit urinary diversion, there is often no obstruction of the ureter. Without hydronephrosis, nephrostomy is deemed to be a difficult procedure, and it is not very safe for patients following radical cystectomy/pelvic exenteration and ileal conduit urinary diversion. Therefore, this procedure should be performed only in high-volume centers by experienced surgeons. Retrograde stenting is much safer than nephrostomy, but exploration of the ureteral anastomosis is time-consuming, and mucosal edema of the ileal conduit and ureter makes the procedure difficult. Additionally, this process has a potential risk of invasive abdominal infection.

Compared to endourology approaches and transperitoneal surgery, the intra-conduit NPS is a more mini-invasive and convenient approach [9, 18]. It is a bedside procedure, but we can’t see the details of the conduit during this process, and the placement of the silicon tube might not be deep enough for certain reasons. Additionally, this procedure should be performed by a surgeon who is familiar with the operation details for each patient. If this procedure is not successful, further management, such as ureteral stenting and/or nephrostomy and even surgery might be needed. Moreover, for 8 patients, the ureteral stent was not removed when urinary fistula was diagnosed. Therefore, retrograde ureteral stenting might not be very reliable for some patients.

Although there is no recommendation of the NPS in the treatment of urinary fistula following ileal conduit urinary diversion, the NPS has often been used in complicated wounds, and its uniform negative pressure can enhance wound healing. In some complicated cases of urine leakage, the NPS was associated with a favorable outcome [15, 16]. In this study, the use of the intra-conduit NPS also resulted in a favorable outcome as a conservative procedure. Therefore, the NPS might be a good alternative for curing urinary fistula following ileal conduit urinary diversion. Compared to other approaches, the intra-conduit NPS is a mini-invasive and compatible approach, and caregivers should attempt its use in clinical practice in selected patients.

This retrospective study did not strictly define the indications of the NPS, and selection bias was inevitable because all patients were in good conditions when they chose the intra-conduit NPS as a conservative treatment for urine leakage. In terms of the rarity of urine leakage, the population was limited, and no control group was recorded. As the NPS is a new approach for treating urine leakage following ileal conduit urinary diversion, advanced studies and long-term follow-up periods are needed. All of these patients were reviewed in our single center, a university-affiliated hospital. Furthermore, these patients received good supportive treatment and consistent observation and evaluation. To our knowledge, this is the largest report of the use of the NPS for urinary fistula. As a conservative treatment, the intra-conduit negative pressure system is a mini-invasive and compatible approach for selected patients with urine leakage following ileal conduit urinary diversion.

## Conclusion

The intra-conduit negative pressure system is a feasible and promising way to cure urinary fistula following ileal conduit urinary diversion. As this system is a mini-invasive and simple approach, it might represent an alternative for nephrostomy in selected patients. Further advanced studies are needed.

## Supplementary information

**Additional file 1.** The installation of negative pressure system

## Abbreviations

IC: Ileal conduit
NPS: Negative pressure system
