# Supplementary figures and images for: Conservative treatment for urinary fistula following ileal conduit urinary diversion: a simple method
Source: BMC Urol. 2019 Dec 10;19:131. doi: 10.1186/s12894-019-0564-3 (PMC6905099; doi:10.1186/s12894-019-0564-3)

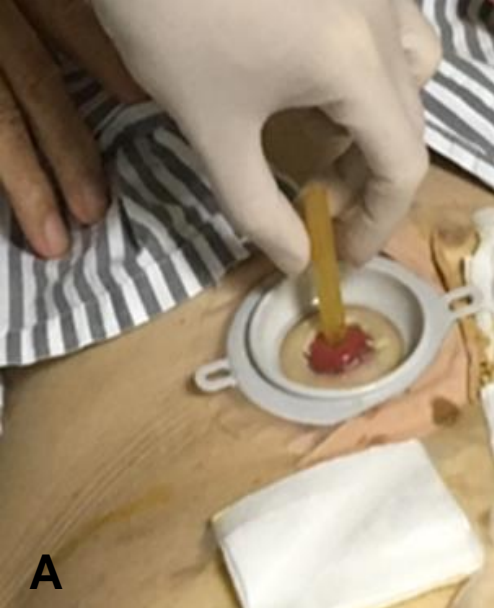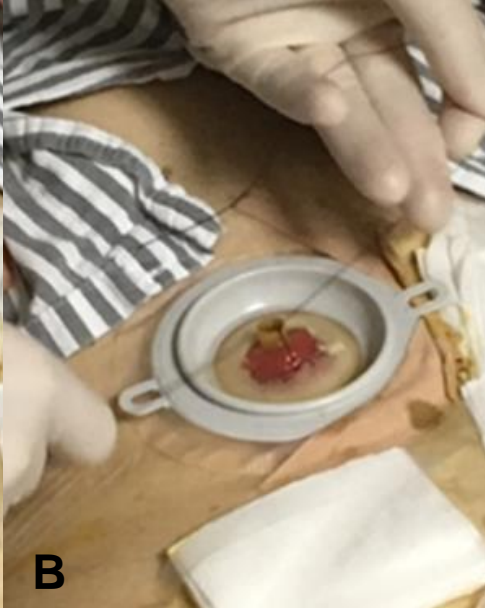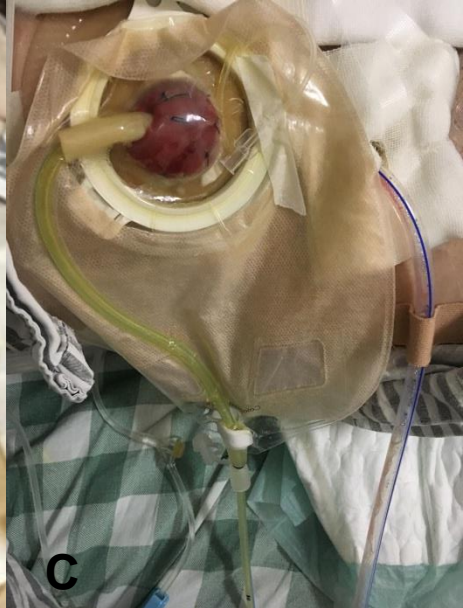

Supplement: Supplementary file 1 — Additional file 1. The installation of negative pressure system [file 12894_2019_564_MOESM1_ESM.pdf]
